# Long-tailed macaques: an unfairness model for humans

**DOI:** 10.1080/19420889.2022.2070902

**Published:** 2022-05-10

**Authors:** Dwi Atmoko Agung Nugroho, Dondin Sajuthi, Sri Supraptini Mansjoer, Entang Iskandar, Huda Shalahudin Darusman

**Affiliations:** a Primatology major, Graduate School Program, IPB University-Indonesia, Bogor City, Indonesia; b Primate Research Center, Institute of Research and Community Service (LPPM), IPB University-Indonesia, Bogor-Indonesia; c Faculty of Veterinary Medicine, IPB University-Indonesia, Bogor-Indonesia

**Keywords:** Equity aversion, ratio, food type preference, long-tailed macaques

## Abstract

The current study was designed to predict why human primates often behave unfairly (equity aversion) by not exhibiting equity preference (the ability to equally distribute outcomes 1:1 among participants). Parallel to humans, besides inequity aversion, lab monkeys such as kin of long-tailed macaques *(Macaca fascicularis)* also demonstrate equity aversion depending on their preference for the outcome (food) type. During the pre-experiment phase, a food-preference test was conducted to determine the most preferred income per individual monkey. Red grapes were the most preferred outcome (100%) when compared to vanilla wafers (0%). The first set of experiments used a 1:1 ratio (equity condition) of grape distribution among six kin-pairs of female long-tailed macaques, and we compared their aversion (Av) versus acceptance (Ac). In the second experiment, we assessed the response to the 0:2 and 1:3 ratio distribution of grapes (inequity condition). A total of 60 trials were conducted for each condition with N = 6 pairs. Our results show aversion to the inequity conditions (1:3 ratios) in long-tailed macaques was not significantly different from aversion to the equity conditions (1:1 ratios). We suggest that the aversion observed in this species was associated with the degree of preference for the outcome (food type) offered rather than the distribution ratio. The subjective preferences for outcome types could bring this species into irrationality; they failed to share foods with an equal ratio of 1:1.

## 1 Introduction

Were primates programmed to have a sense of fairness? It was not reasonable to claim that human primates have a sense of fairness just because they demonstrate a disadvantageous inequity aversion (a protest due to having less than others) as reported in [1], but at the same time deny that they may also demonstrate an aversion to equity [2]. In humans, disadvantageous inequity aversion was considered a sense of fairness 3, but that did not equal equity preference (the ability to split outcomes equally 1:1 among participants). So, the sense of fairness in human primates could be unreliable or just a minor number when they tend to ignore equal distribution ratio (1:1) upon a selfish reward. Moreover, advantageous inequity aversion (a protest toward unequal outcome distribution by advantaged participants) did not always exist in humans [4]. The other consideration was a causal-effect relationship that inequity aversion occurs by inequity condition; inequity condition occurs by an aversion to equity itself. In natural life, inequity aversions happen to respond to inequity conditions, inequity conditions happen due to aversion to equity conditions. This research was to investigate whether this typically human trait was found also in a non-human primate and suggests it has deep roots in human evolution.

Much of the current literature on whether primates (human and non-human) have a sense of fairness focuses on the aversion to the inequitable outcome as a determination that the condition was unfair. [5] showed that capuchin monkeys *(Cebus apella*) demonstrate a sense of fairness by rejecting unequal outcomes, but the behavior observed may have been an expression of frustration [6]. The capuchin monkeys may have rejected the cucumber (one of the food types) because they did not like it, not because their partner got a better food outcome (grape). In other studies, capuchin monkeys failed to show the same inequity aversion when the difference in preference between the two types of food offered was not as significant [7,8]. Silberberg et al. (2009) [7] used pine seeds versus sunflower seeds, and [8], used two types of cereal that were similar. This suggests that capuchin monkeys threw away the cucumber because motivated by a better reward (grape). We should aware that monkeys were rejecting the low-value food (cucumber) because of grapes. Cucumber could be high-value food when versus stone. Previous studies should give cucumber after monkeys receive an equal amount of grapes, monkeys of course will reject cucumber at all. Sense of fairness was not reliable in Brosnan’s experiment, except when the disadvantaged capuchin got a worse food outcome (cucumber), the advantaged partner should have rejected the better food outcome (grape) as an expression of advantageous inequity aversion. But, the degree of preference for the resource itself (most often food) was selfishness to shift the balance toward resource acquisition and away from social benefit. We rarely saw a subject protesting at the advantage. In the context of inequity conditions, often protests only come from subjects who were disadvantaged, while those who were advantaged tend to deny the inequity.

Whether primates exhibit a sense of fairness should also be examined in the context of equity acceptance or aversion [2,4]. In humans, when socially acceptable actions provide one player with a greater portion of an outcome, then the subject will put forth an extra effort to secure the benefit [2]. This behavior persists, even when it was to the detriment of the other players, thus demonstrating a preference for selfishness toward the available outcome. Socially despotic monkeys, such as long-tailed macaques, also did not share preferred rewards to benefit other subjects [9] though they may compare their portion to what others receive [10]. Food competition was a part of social comparison processes in long-tailed macaques [11].Undeniable, some studies have found that monkeys may prefer to equal option 1:1, meaning they could accept equity rather than the inequitable condition [12–15]. Amici et al. (2012) even demonstrated that 76% of dominant long-tailed macaques would accept an equitable distribution (only 24% refusal) when pairs were positioned face to face. However, [16] Schaub (1996) failed to demonstrate kin altruism in long-tailed macaques in a food-sharing experiment, which brings into doubt whether long-tailed macaques would accept the equity 1:1 without another conditioning to predispose them to this behavior.

Our alternative hypothesis was that long-tailed macaques will protest an equitable food distribution (1:1) due to their preferred level for the food being offered. Protest will be apparent by observation of avoidance behavior (throwing the food’s tray without taking the food) or even stealing by taking the partner’s food within the pair. The rationale was that other studies that use test foods other than fruit can potentially fail to demonstrate aversion [7,8].

To test this hypothesis, we conducted two kinds of behavioral experiments with a new paradigm: an equity aversion test (1:1) and inequity aversion tests (2:0 and 1:3). A food-preference test was also conducted as a pre-experimental phase to determine the absolute preferred outcome for the aversion tests. We used a quantity paradigm (number of food distribution) rather than a qualitative paradigm (the type of food distribution) for our aversion tests because the preference for food types was unstable [17]. We expect to show that a zero preference level for food or the lowest food preference (such as vanilla wafer) would not produce negative responses (aversions) at all versus the highest food preference (such as grape) would potentially produce negative responses (we call it as aversion). Sure, food type preferences were unstable when we see monkeys did not averse to equity of grape distribution. This means that the diminished food preference (such as grape) would not potentially produce negative responses. Monkeys would not care about quantity over the quality of food. But the opposite, if monkeys found food they like, of course, monkeys would take that food as much possible as they like without counting again. They just grab whatever they like including grapes without an account for it. Moreover, they would not care about the number of vanilla wafers when they have no function for monkeys (such as monkeys were not hungry from monkey chows). Sure, it would be different if we give vanilla wafers when these monkeys were hungry, monkeys would eat all wafers too.

In the first aversion test, food was distributed using a 1:1 ratio (equity condition) among six pairs of female long-tailed macaques. We recorded responses as aversion (number of food pieces taken was less than the number distributed to the subject, ignoring the food or throwing the tray to maximize its rejection, or when the subject also takes their partners food to maximize their preference) versus acceptance of the condition (where both of subjects in a pair could share food equitably, 1:1). In the second aversion test, food was distributed while using the inequity condition (0:2 and 1:3 ratios) among six female long-tailed macaques, we observed and compared inequity aversion (where there was a subject in a pair did not take the food outcome or were in a pair, the partner’s food was taken to maximizes their aversion) versus equity aversion in the first experiment. One of the weaknesses of 15 was that they did not account for stealing behavior (taking a partner’s food) but only refusal behavior as aversion (negative responses), while Fershtman et al. (2012) [2] demonstrate equity aversion in humans by showing the subject will put forth an extra effort to secure the benefit even when it was to the detriment of the other players. Please note that we have added stealing behavior [taking a partner’s food] to our aversion criteria in modeling human aversion of [2].

**Figure 1.**
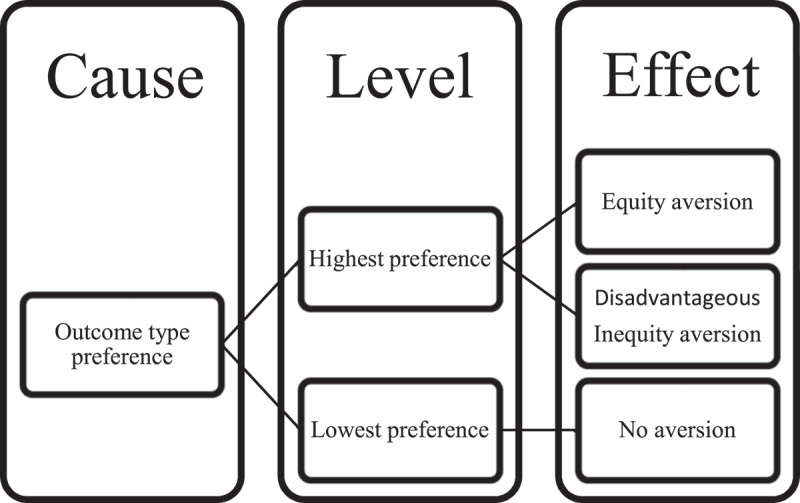
The aversion mechanism was predicted by preferences. The hypothesis was the highest preference level of outcome types would produce both equity and disadvantageous inequity aversion, but the lowest preference level of outcome types would produce no aversion.

This research hypothesis was that their equity aversion would be greater than the equity acceptance due to the absolute preference toward a type of outcome (see Figure 1). Monkeys would be averse to the equity condition (1:1) and not different than the inequity (1:3) condition.

## 2 Methods

### 2.1 Participants

Six female long-tailed macaques *(Macaca fascicularis)* taken from the same troop were used in this study. They were considered as sister subjects due to living together for two years. The animals weighed 3.05 kg±0.187 and ranged 5.75 ± 0.187 years of age. None of the animals had any history of physical or behavioral abnormalities. They have an average arm length of 25 cm±3.416 (N = 6). This study was conducted at the Association for Assessment and Accreditation of Laboratory Animal-accredited Primate Research Center at IPB University, Bogor, Indonesia.

### 2.2 Ethics statement

All animal protocols used in these studies were approved by the Institutional Animal Care and Use Committee of IPB University with Animal Care and Use Committee No. IPB PRC-18-B006. Principles for the ethical treatment of animals in the research environments are described in Government Regulation of Republic of Indonesia No. 95 2012. Specific and detailed directions for the care and use of animals in this research are available in the guidelines, developed in 2011 by the Health Research Ethics Committee, in the Ministry of Health (National Guidelines on Health Research Ethics [18]), and Teaching Guide Book for Ethics on Health Research [19].

### 2.3 Apparatus

**Figure 2.**
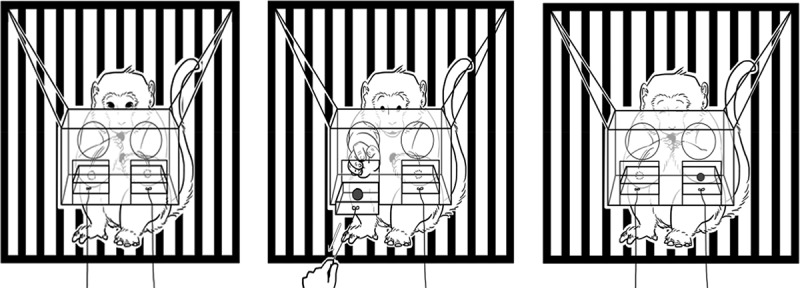
The food-preference test. A monkey making choices between two kinds of foods.

**Figure 3.**
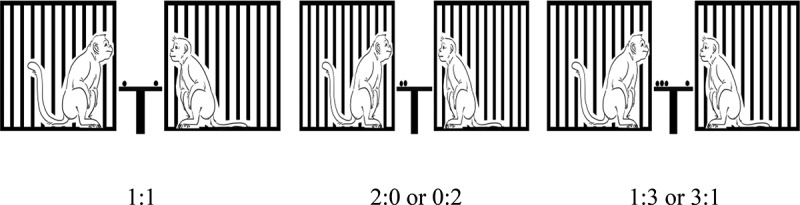
Aversion test. Pairs of a monkey got red grape distributions between them with 1:1, 0:2 or 2:0, and 1:3 or 3:1 ratios.

Six subjects were placed and separated into six individual cages. The size of a cage was 61 × 67 × 88 cm (length × width × height). Distances between bars of each cage were about ±2 cm. For the outcome type preference test, a single piece of food that differed in type was placed into a 16 × 10 × 1-cm tray made from clear acrylic (see Figure 2). For the aversion test, food outcomes were placed on 16x10x1cm of an opened-acrylic tray between cages of pairs with ±50 cm height from the bottom of the floor by an acrylic buffer (see Figure 3). We recorded all responses by using a Canon A2300 video recorder placed at ±50 cm in front of the cages.

### 2.4 Procedures

#### 2.4.1 Habituation

The experimenter started observing these monkeys in a group enclosure for one month. During the observation time, their food intakes toward some foods (red grape, banana, and vanilla wafer) were observed as a criterion to joining them into their next subjective’s preference test toward these outcomes (food) types. After one month, they were transported from the group enclosure into individual cages to joining the outcome types preference test. The animals’ diet consisted of fruit and standard monkey chow pellets provided twice a day. Tap water was provided *ad libitum* throughout the experiment. Subjects were not deprived of monkey chow during testing. Monkeys live in individual cages for only one day in the preference test for all monkeys and 3 days in the aversion test per pair. As long as our vets here realized these monkeys did not stress, we run our procedures.

#### 2.4.2 Food preference test

A food-preference test was conducted to seek the absolute preferred outcome in each monkey. We applied the first-choice test between: a) pieces of the ½ red grape versus banana and b) pieces of the ½ red grape versus pieces of the vanilla wafer in 60 trials (10 trials per subject). The size of ½ of red grape was 1 × 0.5 cm (diameter × height), the size of banana slice was 1 × 0.5 cm (diameter × height), and the size of 1 vanilla wafer was 2 × 2 × 0.5 cm (length × width × height). In each trial, the first chosen food was a representation of the greatest preferred outcome. We choose the red grape as used in Brosnan’s capuchin [20], the banana [21], and the vanilla wafer as a substitution of the monkeys’ daily raisin (Amici et al., 2012). We choose ½ size of the red grape rather than a full size of a red grape to decrease satiation effects on monkey’s rejection within food intakes.

We used the ‘counterbalance’ technique when delivering two kinds of foods in front of the monkey. Such as randomizing sequences food A (left) to B (right) then B (left) to A (right). This technique was addressed to avoid hand or side preference as well. A cocktail spoon was used to ensure size equivalence (1 × 0.5 cm in diameter and depth, respectively) among the two fruits (red grape and banana) considered as prospective paired choice alternatives in 10-trial pretest sessions. The sole criterion for accepting a food pair for the main experimental condition was that both alternatives were sampled during a pretest session. During the test, choices were only permitted to a single alternative. When the subject reached for both foods, the food containers were pulled out of the subject’s reach by the experimenter, who stood approximately 3 m away from the front of the test chamber. When a single food was chosen, the second food was immediately pulled away from the chamber.

Randomized pairs of foods were placed one in each hand and shown to an animal from about a meter’s distance for approximately 5 seconds to allow the animal to visualize the two foods. We then moved the foods within reaching distance and allowed the animal to choose one food item in this two-alternative choice test. The unselected item was removed. We then recorded whether the monkey consumed the food, partially consumed it, or discarded it. When discarded, we waited approximately 1 minute and began another trial. We performed one trial per day and presented each combination of foods 10 times for each monkey. Testing typically occurred after daily feeding [22].

All monkeys fear human experimenters as wild animals still, so they would not take the food till the experimenter goes outside the room after distributing the food in front of them. Each monkey participates only in one session on the same day in all experiments to avoid the satiation effect. They moved from a group enclosure to individual cages, so they knew each other and have social contact experiences. Each cage used a visual barrier at the right and left side. We applied the Five Freedoms which were globally recognized as the gold standard in animal welfare, encompassing both the mental and physical well-being of animals; they include freedom from hunger and thirst; freedom from discomfort; freedom from pain, injury, and disease; freedom to express normal and natural behavior. We keep their natural behavior as wild animals and minimize their stress by making a distance from monkeys. Having no fear of humans was not a natural behavior for monkeys. As wild animals, monkeys naturally fear humans as strangers.

#### 2.4.3 Aversion test

In the context of the study, subject pairs were positioned face to face and then treated with the food distribution. Subjects were divided to make up 6 pairs: pair 1 (subject 1 vs. subject 2), pair 2 (subject 2 vs. subject 3), pair 3 (subject 3 vs. subject 4), pair 4 (subject 4 vs. subject 5), pair 5 (subject 5 vs. subject 6), and pair 6 (subject 6 vs. subject 1). Distances between the cages of pairs were only about 16 cm. We conducted the first experiment by applying the food distribution with a 1:1 ratio only, and the second experiment with 0:2 and 1:3 ratios in 10 trials per pair for each ratio. We treated subject pairs with the order 1-3-5-2-4-6. The experimenter stands out of the pairs’ room after giving the food. In the equity trials, the experimenter gave the first food to actor subjects and then to the partner in 2 seconds by 5-time repetitions, and then gave the first food to partner subjects and then to the actor in 2 seconds by 5-time repetitions. In inequity trials, the experimenter gave the first food to the disadvantageous subject first (for 1:3 and 3:1 ratio) or gave nothing (in 0:2 and 2:0 ratios) and then gave the food to the advantageous subject within 10 seconds. The duration of each trial was about 30 seconds.

We concern if any fear factors such as social anxiety in the social experiment context when responding to both equity and inequity conditions. For sure, refusing a high-value food such as grape was avoidance of the social conditions (such as partner) but not the food. It would be different than refusing a low-value food such as a vanilla wafer means avoiding the food itself rather than the social conditions. We should remind that the manifestation of aversions did not always refuse the food but it could be throwing the tray and stealing too. We designed monkeys were not performing a task to earn the food outcomes but instead were simply given the outcomes rewards due to we expect to observe throwing the tray and stealing behavior.

The monkeys were required to take the outcome that was presented sequentially, positioned 1-cm from a reference point at one side of the tray (see Figure 3). The trial began when the experimenter pressed the 2-cm manual switch on the video recorder with his left hand. The trial was located within the upper reach of the video recorder. After a prestimulus period of 5 seconds, the first outcome was to be distributed above one side (left or right side) of the tray. A delay of 1.0 seconds separated the presentation of the 1st outcome (for the one of monkeys) from the appearance of the 2nd outcome (for the partner), which also lasted 1.0 seconds. The monkeys had to take the stimulus from the reference point within 10 seconds by using their left or right arms outside of the cages. The variable intertrial period was usually around 10 seconds. The duration of the prestimulus periods and delays and the position of the stimuli were pseudorandomly determined (see Table 1).

**Table 1.** Aversion test stimulus procedure

Ratio	∑ food		N	Duration (Second)	Interval (Second)
	S(x)	S(y)			
1:1	1	1	10	30	10
0:2	0	2	5	30	10
2:0	2	0	5	30	10
1:3	1	3	5	30	10
3:1	3	1	5	30	10

Index: S(x) = Actor, S(y) = partner, N = repetition

Experiment was done in 6 days. Day 1^st^: pairs 1-3-5→1:1, 1:1, 1:1, 1:1, 1:1, 1:1, 1:1, 1:1, 1:1, 1:1. Day 2^nd^: pairs 2-4-6→1:1, 1:1, 1:1, 1:1, 1:1, 1:1, 1:1, 1:1, 1:1, 1:1. Day 3^rd^: pairs 1-3-5→ 0:2, 0:2, 0:2, 0:2, 0:2, 2:0, 2:0, 2:0, 2:0, 2:0. Day 4^th^: pairs 2-4-6→ 0:2, 0:2, 0:2, 0:2, 0:2, 2:0, 2:0, 2:0, 2:0, 2:0. Day 5^th^: pairs 1-3-5→1:3, 1:3, 1:3, 1:3, 1:3, 3:1, 3:1, 3:1, 3:1, 3:1. Day 6^th^: pairs 2-4-6→ 1:3, 1:3, 1:3, 1:3, 1:3, 3:1, 3:1, 3:1, 3:1, 3:1. We gave one day of rest per pair between conditions to erase the inter-conditioning influence. We did not give more counterbalanced orders per pair due to we did not want to give them more stressful events. We were always keeping the animal welfare of these monkeys.

**Figure 4.**
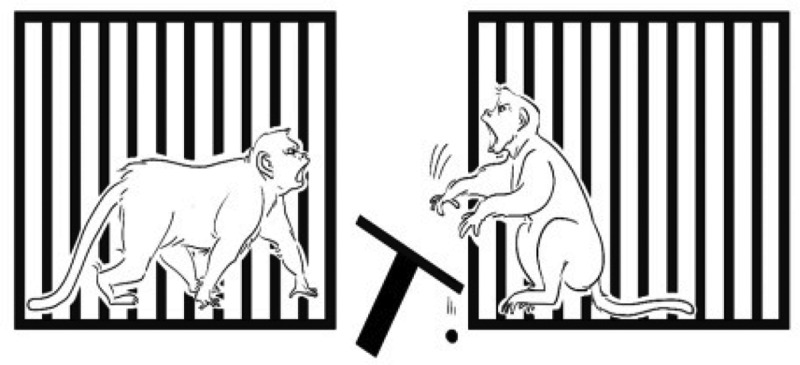
Avoidance. A monkey did not take the food but throwing the tray to maximize its rejection.

**Figure 5.**
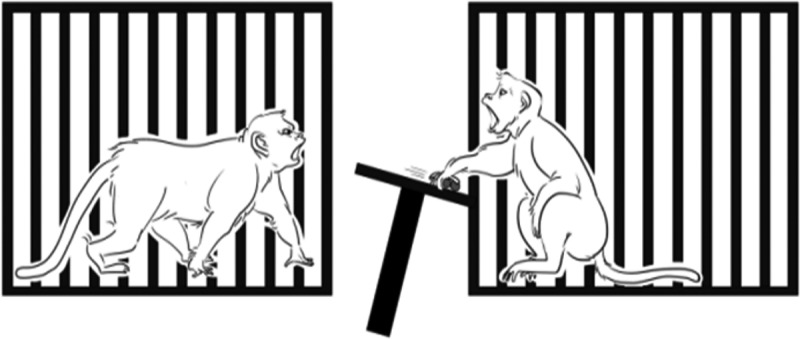
Stealing. A monkey taking the partner’s outcome to maximize its preference.

In general, aversion was defined by all negative responses including avoidance (did not take the food, throwing the tray) and stealing the partner’s food. We define Aversion (Av) as one of the criterions such as a) Avoidance which was when the number of food intake of a monkey was less than food distribution but just going away from the condition or a monkey did not take the food but threw the tray to maximize its rejection (see Figure 4) not due to be stolen by its partner, b) Stealing which was when the amount of food intake was more than food distribution due to a monkey taking the partner’s food to maximize its preference (see Figure 5). We define Acceptance (Ac) as when the food intake was the same as the food distribution. Based on these definitions, we measured the rate and percentage of each behavior.

Avoidance criteria were adopted from refusal behavior by Amici et al. (2012), but please note that we disagree with researchers who did not see stealing behavior (taking others’ part) as an aversion. We define stealing as an aversion (a negative response) too. We have a standard in this case; taking others’ part was a problematic social behavior (negative behavior). Comparable to the monkey behavior context, we stick to our basic scientific standards of problematic social behavior that:

1. The first condition was when an individual monkey was given two foods, and then it grabs all two foods. This was a normal behavior (positive behavior).
2. In the second condition, when two monkeys were given two foods, and then a monkey grabs all these two foods. This was a problematic social behavior (negative behavior).
3. The reason was that the first condition was an individual context, but the second was a social context. We call a healthy monkey in the first condition, but an irrational monkey for a monkey who fails to share outcomes by a 1:1 ratio with a partner in the second condition.
4. Based on the social context of this aversion problem (two monkeys facing each other), in the analysis section of the aversion test, we used paired analysis as the unit of analysis rather than individual analysis.

#### 2.4.4. Stimuli availability

All stimuli videos and all of the materials used to conduct the current research have been included as supplemental materials.

### 2.5 Data analyses

#### 2.5.1 Food preference test

A simple descriptive data of frequencies, mean ($\bar{X}$), Standard Deviation (SD), p-value, number of the subject (N), and percentage of first-choice food in 60 trials with a binominal test was presented to seek the absolute preferred outcome in these monkeys. A dependent-samples t-test was conducted to compare the choice between foods. An additional two-sided test of Fisher’s exact test was used for confirmation.

#### 2.5.2 Aversion test

A simple descriptive data of frequencies and percentage of aversion and acceptance in 60 trials with the binominal test was presented. Aversion in a pair was the number of trials minus acceptance. Pair’s Av = n-Ac. Then a total of the number of responses (Σx), mean ($\bar{X}$), Standard Deviation (SD), number of subjects (N), and variance (s^2^) accounted for 60 trials (a 10-trial × 6 pairs). Independent Samples t-Test was used to compare Aversion (Av) versus Acceptance (Ac) under 1:1 conditions. Then, a Repeated-Measures ANOVA was used to compare Aversion (Av) between equity versus inequity conditions such as a) 1:1 versus 0:2 (and 2:0) condition, b) 1:1 versus 1:3 (and 3:1) condition and, c) 0:2 (and 2:0) versus 1:3 (and 3:1) condition. An additional two-sided test of Fisher’s exact test and chi-square test were used for confirmations.

## 3 Results

### 3.1 Food preference test

**Figure 6.**
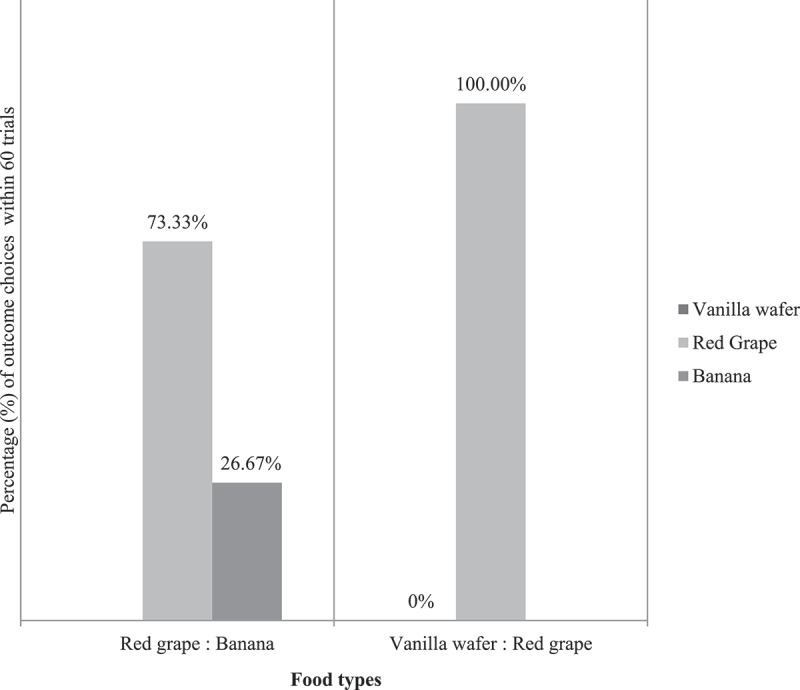
Percentage (%) of outcome choices between vanilla wafer, red grape, and banana within 60 trials (10 × 6 subjects). These outcome type preference test results show that the red grape was the absolute preferred outcome (100%) compared to the vanilla wafer (0%) and it was still a higher preferred outcome (73.33%) compared to the banana (26.67%).

We found that the preference for the piece of ½ red grape versus a slice of banana in 60 trials (10 trials × 6 subjects) was 44/60 (7.3 ± 4.32, p = 0.001, N = 6 subjects): 16/60 (2.7 ± 4.32, p = 0.999, N = 6 subjects), and the piece vanilla wafer versus the piece of ½ red grape in 60 trials (10 trials × 6 subjects) were 0/60 (p = 0.999): 60/60 (p < 0.001), so we chose the ½ red grape as the absolute preferred-outcome (100%) for the next aversion test (see Figure 6). A dependent-samples t-test was conducted to compare the choice between a banana and a red grape. There was a significant difference in the preference for banana (M = 1.6, SD = 0.516, N = 6 subjects) and red grape (M = 4.4, SD = 0.516, N = 6 subjects) conditions; t (18) = −12.124, p < 0.05. Based on a two-sided test of Fisher’s exact test, red grape are more likely than banana X^2^ (1, N = 6 subjects) = 26.133, p = <0.001 with p < 0.05. Red grape was more likely than vanilla wafer (p = <.001) with p < 0.05. These results suggest that food types do affect preference. Specifically, our results suggest that the preference for red grape was higher than a banana and a vanilla wafer in these monkeys.

### 3.2 The aversion test

In 60-trials of the equity condition, we found that Acceptance (Ac) under 1:1 conditions of ½ red grape distribution was 12/60 (20%) (p = 0.999), and Aversion (Av) was 48/60 (80%) (p = 0.001). Aversions were included (7/60) 11.67% avoidances and (41/60) 68.33% stealing behaviors. An Independent Samples t-Test was used to compare Aversion (Av) versus Acceptance (Ac) under 1:1 conditions of ½ red grape distribution. The paired-samples t-tests were used to make post hoc comparisons between conditions. The paired samples t-test indicated that there was a significant difference in the scores for Aversion (M = 8, SD = 2.280, N = 6 pairs) and Acceptance (M = 2, SD = 2.280, N = 6 pairs) conditions; t(10) = 4.557, p < 0.05. A chi-square test of independence was performed to examine the relation between grape preferences and equity aversion. The relation between these variables was significant, X^2^ (1, N = 6) = 15, p = 0.000108, p < 0.05. Equity aversions were more likely than acceptance. Sure, nothing to do with aversion when we conduct the same procedure by using vanilla wafers due to we found 0% of stealing behavior. These results suggest that outcome type preferences did have any effect on equity aversion. Specifically, our results suggest that when monkeys have the highest preference for a food type such as red grape (100%), they would be averse to equity conditions significantly rather than when they have the lowest preference for a food type such as vanilla wafer (0%).

In 60-trials of inequity conditions (0:2), we found that Acceptance (Ac) under 0:2 conditions of ½ red grape distribution was 22/60 (36.67%) (p = 0.999), and Aversion (Av) was 38/60 (63.33%) (p = 0.001). Stealing behaviors were 32/60 (53.33%). There 6/60 (10%) were advantage-avoidances in 0:2 conditions but not significant (p = 0.999).

In 60-trials of inequity conditions (1:3), we found that Acceptance (Ac) under 1:3 conditions of ½ red grape distribution was 9/60 (p = 0.999) (15%) and Aversion (Av) was 51/60 (85%) (p = 0.001). Aversions were included (8/60) 13.33% disadvantage-avoidances and (38/60) 63.33% stealing behaviors. There 5/60 (8.33%) were advantage-avoidances in 1:3 conditions but not significant (p = 0.999).

A one-way within-subjects (or repeated measures) ANOVA was conducted to compare the effect of ratio type on the number of Aversions (Av) in 1:1 (equity) versus 0:2 (inequity) and 1:1 (equity) versus 1:3 (inequity) conditions of red grape distribution. Two paired samples t-tests were used to make post hoc comparisons between conditions. There was no significant effect of 1:1 versus 0:2 ratio types on Aversion, Wilks’ Lambda = 0.10, F(1,4) = −0.108, p = 0.267, p > 0.05. A paired samples t-test indicated that there was no a significant difference in the scores of Aversion for 1:1 ratio (M = 8, SD = 2.280, N = 6 pairs) and 0:2 ratio (M = 6.33, SD = 2.160, N = 6 pairs) conditions; t(5) = 1.250, SD = 1.333, p = 0.267, at p < 0.05. There was no significant effect of 1:1 versus 1:3 ratio types on Aversion, Wilks’ Lambda = 0.10, F(1,4) = −0.107, p = 0.665, p > 0.05. A paired samples t-test indicated that there was no a significant difference in the scores of Aversion for 1:1 ratio (M = 8, SD = 2.280, N = 6 pairs) and 1:3 ratio (M = 8.5, SD = 1.224, N = 6 pairs) conditions; t(10) = 0.4733, SD = 1.056, p = 0.646, at p < 0.05.

Based on a two-sided test of Fisher’s exact test, we found that there was no significant difference in the scores of equity aversion (1:1) versus inequity aversion (0:2) with p = 0.067, p < 0.05, N = 6 pairs. A chi-square test of independence was performed to examine the effect of 1:1 versus 0:2 ratios on aversion. The differences affect between these ratios were little significant, X^2^ (1, N = 6) = 4.104, p = 0.043, p < 0.05. Aversions in 1:1 ratios were a little more likely than in 0:2 ratios.

Based on a two-sided test of Fisher’s exact test, we found that there was no significant difference in the scores of equity aversion (1:1) versus inequity aversion (1:3) with p = 0.631, p < 0.05, N = 6 pairs. A chi-square test of independence was performed to examine the effect of 1:1 versus 1:3 ratios on aversion. The differences affect between these ratios were not significant, X^2^ (1, N = 6) = 0.519, p = 0.471, p < 0.05. Aversions in 1:3 ratios were not more likely than in 1:1 ratios.

A chi-square test of independence was performed to examine the effect of 0:2 versus 1:3 ratios on aversion. The differences affect between these ratios were significant, X^2^ (1, N = 6) = 7.350, p = 0.0067, p < 0.05. Aversions in 1:3 ratios were more likely than in 0:2 ratios.

These results suggest that ratios almost did not have a great effect on Aversion (Av) since we found no significant effect between 1:1 versus 1:3 ratios. Our results suggest that there was no significant difference in the scores of equity (1:1) versus inequity aversion (1:3) caused by grape preference. The complete data could be seen in Tables 2-5.

**Table 2.** The number of food intakes per monkey per trial in a pair (10 trials per pair × 6 pairs) to 1:1 ratio (equity condition)

Trial	Outcome ratio	Number of food intakes per monkey in pairs											
		M1 vs M2		M2 vs M3		M3 vs M4		M4 vs M5		M5 vs M6		M6 vs M1	
1	1:1	M1 = 0	M2 = 2	M2 = 2	M3 = 0	M3 = 0	M4 = 2	M4 = 0	M5 = 2	M5 = 2	M6 = 0	M6 = 0	M1 = 2
2	1:1	M1 = 0	M2 = 2	M2 = 2	M3 = 0	M3 = 0	M4 = 2	M4 = 1	M5 = 1	M5 = 2	M6 = 0	M6 = 0	M1 = 1
3	1:1	M1 = 0	M2 = 2	M2 = 2	M3 = 0	M3 = 0	M4 = 2	M4 = 0	M5 = 1	M5 = 1	M6 = 1	M6 = 0	M1 = 1
4	1:1	M1 = 0	M2 = 2	M2 = 1	M3 = 1	M3 = 0	M4 = 2	M4 = 0	M5 = 2	M5 = 0	M6 = 2	M6 = 1	M1 = 1
5	1:1	M1 = 2	M2 = 0	M2 = 2	M3 = 0	M3 = 0	M4 = 2	M4 = 0	M5 = 2	M5 = 2	M6 = 0	M6 = 1	M1 = 1
6	1:1	M1 = 2	M2 = 0	M2 = 2	M3 = 0	M3 = 0	M4 = 2	M4 = 0	M5 = 2	M5 = 2	M6 = 0	M6 = 2	M1 = 0
7	1:1	M1 = 0	M2 = 2	M2 = 2	M3 = 0	M3 = 0	M4 = 2	M4 = 0	M5 = 2	M5 = 1	M6 = 1	M6 = 1	M1 = 1
8	1:1	M1 = 2	M2 = 0	M2 = 2	M3 = 0	M3 = 0	M4 = 2	M4 = 0	M5 = 1	M5 = 2	M6 = 0	M6 = 1	M1 = 1
9	1:1	M1 = 0	M2 = 2	M2 = 0	M3 = 1	M3 = 0	M4 = 2	M4 = 0	M5 = 1	M5 = 1	M6 = 1	M6 = 1	M1 = 1
10	1:1	M1 = 0	M2 = 2	M2 = 1	M3 = 1	M3 = 0	M4 = 2	M4 = 0	M5 = 1	M5 = 0	M6 = 2	M6 = 1	M1 = 1

**Table 3.** The number of food intakes per monkey per trial in a pair (10 trials per pair × 6 pairs) to 0:2 ratio (inequity condition)

Trial	Outcome ratio	Number of food intakes per monkey in pairs											
		M1 vs M2		M2 vs M3		M3 vs M4		M4 vs M5		M5 vs M6		M6 vs M1	
1	0:2	M1 = 0	M2 = 2	M2 = 2	M3 = 0	M3 = 0	M4 = 2	M4 = 1	M5 = 1	M5 = 2	M6 = 0	M6 = 0	M1 = 2
2	0:2	M1 = 0	M2 = 1	M2 = 2	M3 = 0	M3 = 0	M4 = 2	M4 = 1	M5 = 1	M5 = 2	M6 = 0	M6 = 0	M1 = 2
3	0:2	M1 = 1	M2 = 1	M2 = 2	M3 = 0	M3 = 0	M4 = 2	M4 = 2	M5 = 0	M5 = 2	M6 = 0	M6 = 0	M1 = 2
4	0:2	M1 = 2	M2 = 0	M2 = 2	M3 = 0	M3 = 0	M4 = 2	M4 = 0	M5 = 1	M5 = 2	M6 = 0	M6 = 0	M1 = 2
5	0:2	M1 = 0	M2 = 2	M2 = 0	M3 = 2	M3 = 0	M4 = 2	M4 = 0	M5 = 1	M5 = 0	M6 = 0	M6 = 0	M1 = 2
6	2:0	M1 = 2	M2 = 0	M2 = 2	M3 = 0	M3 = 0	M4 = 2	M4 = 0	M5 = 1	M5 = 1	M6 = 1	M6 = 0	M1 = 2
7	2:0	M1 = 2	M2 = 0	M2 = 2	M3 = 0	M3 = 0	M4 = 2	M4 = 0	M5 = 1	M5 = 0	M6 = 2	M6 = 0	M1 = 2
8	2:0	M1 = 0	M2 = 2	M2 = 2	M3 = 0	M3 = 0	M4 = 2	M4 = 0	M5 = 2	M5 = 0	M6 = 2	M6 = 0	M1 = 2
9	2:0	M1 = 2	M2 = 0	M2 = 2	M3 = 0	M3 = 0	M4 = 2	M4 = 2	M5 = 0	M5 = 0	M6 = 2	M6 = 0	M1 = 2
10	2:0	M1 = 0	M2 = 2	M2 = 0	M3 = 2	M3 = 0	M4 = 2	M4 = 2	M5 = 0	M5 = 0	M6 = 2	M6 = 0	M1 = 2

**Table 4.** The number of food intakes per monkey per trial in a pair (10 trials per pair × 6 pairs) to 1:3 ratio (inequity condition)

Trial	Outcome ratio	Number of food intakes per monkey in pairs											
		M1 vs M2		M2 vs M3		M3 vs M4		M4 vs M5		M5 vs M6		M6 vs M1	
1	1:3	M1 = 0	M2 = 2	M2 = 4	M3 = 0	M3 = 0	M4 = 4	M4 = 4	M5 = 0	M5 = 1	M6 = 3	M6 = 0	M1 = 4
2	1:3	M1 = 4	M2 = 0	M2 = 4	M3 = 0	M3 = 0	M4 = 4	M4 = 4	M5 = 0	M5 = 2	M6 = 0	M6 = 0	M1 = 4
3	1:3	M1 = 1	M2 = 3	M2 = 4	M3 = 0	M3 = 0	M4 = 4	M4 = 3	M5 = 1	M5 = 1	M6 = 3	M6 = 0	M1 = 4
4	1:3	M1 = 1	M2 = 3	M2 = 4	M3 = 0	M3 = 0	M4 = 3	M4 = 4	M5 = 0	M5 = 0	M6 = 3	M6 = 0	M1 = 4
5	1:3	M1 = 4	M2 = 0	M2 = 4	M3 = 0	M3 = 1	M4 = 3	M4 = 3	M5 = 1	M5 = 1	M6 = 0	M6 = 1	M1 = 3
6	3:1	M1 = 3	M2 = 1	M2 = 4	M3 = 0	M3 = 0	M4 = 4	M4 = 4	M5 = 0	M5 = 3	M6 = 1	M6 = 0	M1 = 4
7	3:1	M1 = 4	M2 = 0	M2 = 3	M3 = 1	M3 = 0	M4 = 4	M4 = 4	M5 = 0	M5 = 0	M6 = 0	M6 = 0	M1 = 4
8	3:1	M1 = 3	M2 = 0	M2 = 3	M3 = 0	M3 = 0	M4 = 3	M4 = 4	M5 = 0	M5 = 0	M6 = 1	M6 = 0	M1 = 1
9	3:1	M1 = 1	M2 = 0	M2 = 3	M3 = 0	M3 = 0	M4 = 3	M4 = 4	M5 = 0	M5 = 2	M6 = 0	M6 = 0	M1 = 4
10	3:1	M1 = 4	M2 = 0	M2 = 0	M3 = 2	M3 = 0	M4 = 2	M4 = 4	M5 = 0	M5 = 2	M6 = 0	M6 = 0	M1 = 4

**Table 5.** Acceptance vs Aversion to equity (1:1 ratio) and inequity condition (0:2 and 1:3 ratio) per pair in 10 trials

Pairs	1:1		0:2		1:3	
	Acceptance	Aversion	Acceptance	Aversion	Acceptance	Aversion
M1 vs M2	0	10	5	5	3	7
M2 vs M3	2	8	5	5	1	9
M3 vs M4	0	10	5	5	1	9
M4 vs M5	1	9	2	8	0	10
M5 vs M6	3	7	0	10	3	7
M6 vs M1	6	4	5	5	1	9
Σx	12	48	22	38	9	51
$\bar{X}$	2	8	3.7	6.3	1.5	8.5
%	20%	80%	37%	63%	15%	85%
SD	2.280	2.280	2.160	2.160	1.224	1.224
s^2^	5.20	5.20	4.67	4.67	1.50	1.50

**Index**: M = monkey SD = Standard Deviation, N = number of the subject, Σx = number of respond, $\bar{X}$=mean, s^2^ = variance

## 4 Discussions

These data show that accepting the equal number of the grape-outcomes distribution (1:1) was only a minority but not a majority in long-tailed macaques. We suggest that they ignore the ratio upon preferred food. Although we found 20% of acceptance in 1:1 conditions, 10% of advantage-avoidances in 0:2 conditions, and 8.33% of advantage-avoidances in 1:3 conditions, unfortunately, they were not significant enough to support predictions of neither equity preference nor advantageous inequity aversion [4]. This data supports our prediction that using highly preferred resource outcomes (food type) will ensure both equity (1:1) and inequity aversion (1:3) in pairs of long-tailed macaques. These monkeys were averse to the equity condition 1:1 by showing avoidance behavior (without taking the food or even throwing the tray) and stealing (taking more food from their partners) and did not differ from the inequity (1:3) condition. These data did not also support all previous findings that these monkeys preferred the equity over the inequity condition [12–14, Amici et al. (2012), due to showing aversion which did not differ significantly among equity (1:1) and inequity conditions (1:3). Interestingly, the acceptance of the inequity conditions of 0:2 was 36.67%, higher than the acceptance of equity (1:1) conditions at 20% and both ratios have a statistical significance with p = 0.043 for aversion levels. These data support [16], that food-sharing in despotic monkeys such as long-tailed macaques was rare. We only can say that these monkeys need another conditioning to predispose them to accept more equity conditions since this behavior was a minority. Moreover, the acceptance of the inequity conditions of 0:2 was 36.67%, higher than the acceptance of another inequity (1:3) conditions at 15% and both ratios have a statistical significance with p = 0.0067 for aversion levels. These data imply that giving nothing food at all to disadvantaged subjects in 0:2 conditions was not even more provocative rather than giving little outcomes in 1:3 conditions for disadvantaged subjects in these despotic monkeys.

This result should comparable to the finding in humans, that humans did not always accept equity unless conditioned by social norms [2]. This result was different from Amici et al. (2012) that dominant monkeys even accept equity conditions (1:1) around 76% (24% rejections). The results of Amici et al. (2012) differ from the results of [23], that it was subordinate subjects who accept more equity. The results of Amici et al. (2012) raise a new question: when two monkeys facing each other were fed 1:1 then the dominant one takes two foods, was it interpreted that the dominant one fairly behaves just because the stealing behavior was not modeled?

The data made us agree with any findings suggesting that aversion in non-human primates happens by reference to the outcome (food) types [5,20,21,24–28]. We may see the food-sharing of non-human primates would be predicted by nonfood referential such as the degree of dominance as seen in [23], and Amici et al. (2012). The relationship between dominance and hormonal factors may simply explain by cortisol and testosterone concentrations [29], but we suggest that nothing to do with the dominance factor without any reference to the outcome (food) types, due to we should test dominance factor within food-sharing or food competition.

We would not suggest that the aversion level in these monkey’s stealing would be controlled by physiological factors such as the degree of satiation because we did not use a deprivation method in our experiment, our monkeys were not hungry here (daily rations as monkey chow were always available during the experiment). When they did show avoidance behavior then it did not mean that: 1) they did not like the food, because the preference test shows that these monkeys love grapes 100%, or 2) they were full because they could fight for the grape after refusing a condition. Avoidance of the condition was the submissive behavior of despotic monkeys to avoid the pain effect of food competition (Amici et al., 2012). Moreover, compared to rhesus and pig-tailed macaques, long-tailed macaques have a middle level of social anxiety when facing a social stressor to their natural temperament [30]. We should recognize refusing to take food due to fear of retaliation by their partner, but it should remind us of three things: 1) refusing a high-value food such as grape was avoidance of the conditions (retaliation by their partner) but not the food. It would be different than refusing a low-value food such as a vanilla wafer means avoiding the food itself rather than the conditions, 2) the aversion levels of 1:1 versus 1:3 were not significant (p = 0.471), this tells us that monkeys did not care about ratio, they were ignoring ratio whenever they like the type of food (grapes in this respect). This was a great finding since the aim of this study was to answer why monkeys often did not exhibit equity preference (the ability to equally distribute outcomes 1:1 among participants). The answer was that they ignore the ratio of high-value foods.

The limitation of this study was that we could not provide data to show when the lowest preferred food such as vanilla wafers could improve equity acceptance rather than aversion as a logical consequence of the highest preferred food such as red grapes could increase equity aversion rather than acceptance. We can ensure that stealing for vanilla wafers in these monkeys was impossible due to their zero (0%) preferences toward it, but avoidance within vanilla wafer stimulation would equal no giving response at all (neutral). Moreover, due to their zero (0%) preferences for vanilla wafer, measuring their acceptance toward it was impossible. The stealing behavior of vanilla wafer was 0% due to these monkeys did not hungry from monkey chows, and all trials were always done after they got monkey chows. We expect that monkeys always take grapes and always reject vanilla wafers (when grape versus vanilla wafers), it made sense for us due to vanilla wafers were not much different than monkey chows (monkey daily biscuits). So, vanilla wafers have no function when monkeys did not hungry for monkey chows. Monkeys would be eating vanilla wafers only if we deprived them of monkeys chow (their daily biscuits) before trials.

There were other studies predicting food-sharing behavior beyond food preference, such as the role of hormone levels on food-sharing in non-human primates [31,32]. The hormonal-control possibility of food-sharing may come from the ‘oxytocin’ explanation [33], where subjects showed higher urinary oxytocin levels after single food-sharing. Unfortunately, the relationship between food-sharing and oxytocin levels was not reliable too [34]. These kinds of studies convince us that predicting food-sharing behavior beyond food preference was a weakness.

The only hypothesis toward stealing behavior in food-sharing of non-human primates may come from outcome (food) preference, as we suggest that nothing to do with the idea of dominance level before outcome (food) preference. We never saw monkeys would steal vanilla wafers after being given monkey chows like as we did not believe that they would steal stones since stones cannot be eaten equally to vanilla wafers in this respect. Moreover, the social rank in a pair may unstable due to it could change when we saw they could exhibit stealing from one another. For instance here: based on food intakes in the equity condition (1:1), when monkey 4 versus monkey 5, monkey 4 (took only 1 grape) was subordinate to monkey 5 (took 15 grapes). But in the inequity condition (1:3), when monkey 4 versus monkey 5, monkey 4 (took 38 grapes) was dominant over monkey 5 (took only 2 grapes). Again, in the equity condition (1:1), when monkey 1 versus monkey 2, monkey 1 (took only 6 grapes) was subordinate to monkey 2 (took 14 grapes). But in the inequity condition (1:3), when monkey 1 versus monkey 2, monkey 1 (took 25 grapes) was dominant over monkey 2 (took only 9 grapes). So we have no idea if the preference for social rank (dominance) was the first factor related to stealing food before a preference for the outcome (food).

Some studies can successfully demonstrate aversion by using other fruits than grapes such as watermelon, apples, and bananas [17,21,24,26,35]. Whereas the nutritional value and color of the fruits vary, they can produce the same aversion, this meant that the nutritional value and color may not affect the aversion but the level of the monkey’s preference to food types.

Grape energy in this study was 1.035 kcal per piece and the vanilla wafer energy was 20 kcal per piece, which meant that the vanilla wafer energy was higher than a grape. However, grape preferences were 60 times greater than vanilla wafers, which meant that the amount of energy was not directly proportional to the amount of preference. Reluctance to inequity and equity will only occur when the preferred food can be a natural reward [36] that was responded to by striatal neurons in the striatum. Striatal neurons in the basal ganglia of the brain will respond to rewards received by the partner and those received by themselves [37]. Capuchin in [20] gave an aversion response when given a cucumber because the frequency of choice on the preference test shows it likes grape more than a cucumber. Our speculation about this preference was that grape constitutes a lot of pleasure from water and sugar than cucumber [38].

As an analogy, in a human sub-culture that uses money as a bartering tool, the value of money will be contested, while in other cultures that did not use money as a bartering tool, the amount of money value would not be contested. This means that the roots of aversion were not determined by the medium (what) but by the level at which the subject behaves favorably toward the medium (how). In a more universal language, aversion was determined by how much the subject was bound to the material. It can be concluded that the root of aversion lies not in the amount of resource material but in the factor of preference or favoritism toward the resource material.

Based on this research, we suggest that humans evolutionary were not programmed to have a sense of fairness as we show both equity aversion (1:1) and inequity aversion (1:3) occur at the same time in nonhuman primates parallel to humans as their relative closed-species [1,2,4,39,40]. These results like want to show an old saying of Vincent van Gogh: “the more you love the more you suffer”! In other words for these monkeys “the more they prefer the more they averse”! So their “preference” was equal was to “suffer”! Inferior monkeys could avoid both equity and inequity conditions to avoid suffering which was caused by their preference. As an analogy, humans who have preferences for money will have two choices; first: they will fight to get money at any cost, or second: they will avoid the condition because they did not want to be wounded in fighting though they still keep their preference (suffer) to money. It means that inferior humans will also reject the equity and inequity of income distribution when they have a greater preference for such outcome type but felt disadvantaged.

So, we suggest that primates did have not a sense of fairness due to their preference level for a specific outcome could produce their equity aversion (1:1) and would not differ from inequity aversion (1:3). The present research shows that this typically human trait was found also in a non-human primate and suggests that it has deep roots in human evolution. These results suggest that primates maybe not be programmed to have a sense of fairness. Fairness within food sharing was impossible when the individual preference for food types produces social inequity in food intake. The subjective preferences to outcome types may shape both equity aversion and disadvantageous inequity aversion toward partners even with their kin by doing avoidance and stealing. In other words, the subjective preferences to outcome types could bring this species into irrationality; they failed to share foods with an equal ratio of 1:1.

## Data Availability

The authors confirm that the data supporting the findings of this study were available within the article and its supplementary material.

## Highlights

- The idea of fairness based on inequity aversion was unreliable. Inequity aversion did not mean accepting equity. Monkeys could be averse to both inequity and equity conditions at the same time.
- Both inequity and equity aversion could happen due to monkeys did not care about the number of preferred food outcomes.
- Aversion here was defined by all negative responses including avoidance (not taking the food, throwing the tray) and stealing the partner’s food.
- We conclude that a sense of fairness was a minority in primates, and we found that these monkeys’ behavior was comparable with human behavior. Long-tailed macaques were good models of unfairness for humans.

## References

[1] Pabayo R, Molnar BE, Kawachi I. The Role of Neighborhood Outcome Inequality in Adolescent Aggression and Violence. Journal of Adolescence Health. 2014;55(4):571–579. 10.1016/j.jadohealth.2014.04.012 24908384

[2] Fershtman C, Gneezy U, List JA. Equity aversion: social norms andthe desire be ahead. Aej. 2012;4(4):131–144.

[3] Fehr, E., & Schmidt, KM. (1999). A Theory of Fairness, Competition, and Cooperation. The Quarterly Journal of Economics, 114(3), 817–868. 10.1162/003355399556151

[4] Li O, Xu F, Wang L. Advantageous inequity aversion does not always exist: the role of determining allocations modulates preferences for advantageous inequity. Front Psychol. 2018;9:749. 29887817 10.3389/fpsyg.2018.00749 PMC5981801

[5] Brosnan SF, de Waal FBM. Monkeys reject unequal pay. Nature. 2003;425(6955):297–299. 13679918 10.1038/nature01963

[6] Roma PG, Silberberg A, Ruggiero AM, et al. Capuchin monkeys, inequity aversion, and the frustration effect. J Comp Psychol. 2006;120(1):67–73. 16551166 10.1037/0735-7036.120.1.67

[7] Silberberg A, Crescimbene L, Addessi E, et al. Does inequity aversion depend on a frustration effect? A test with capuchin monkeys *(Cebus apella)*. Anim Cogn. 2009;12(3):505–509. 19184138 10.1007/s10071-009-0211-6

[8] McAuliffe K, Chang LW, Leimgruber KL, et al. Capuchin monkeys, *Cebus apella*, show no evidence for inequity aversion in a costly choice task. Anim Behav. 2015;103:65–74.

[9] Sterck EHM, Olesen CU, Massen JJM. No costly prosociality among related long-tailed macaques *(Macaca fascicularis)*. J Comp Psychol. 2015;129(3):275–282. 25961651 10.1037/a0039180

[10] Schmitt V, Federspiel I, Eckert J, et al. Do monkeys compare themselves to others? Anim Cogn. 2016;19(2):417–428. 26615416 10.1007/s10071-015-0943-4 PMC4751161

[11] Keupp S, Titchener R, Bugnyar T, et al. Competition is crucial for social comparison processes in long-tailed macaques. Biol Lett. 2019;15(3):20180784. 30890067 10.1098/rsbl.2018.0784 PMC6451389

[12] De Waal FBM, Leimgruber K, Greenberg AR. Giving is self-rewarding for monkeys. PNAS. 2008;105(36):13685–13689. 18757730 10.1073/pnas.0807060105 PMC2533250

[13] Lakshminarayanan VR, Santos LR. Capuchin monkeys are sensitive to others’ welfare. Curr Biol. 2008;18(21):999–1000. 10.1016/j.cub.2008.08.057 19000809

[14] Massen JJM, van den Berg LM, Spruijt BM, et al. Generous leaders and selfish underdogs: pro-sociality in despotic macaques. PloS ONE. 2010;5(3):e9734. 20305812 10.1371/journal.pone.0009734 PMC2840023

[15] Amici F, Call J, Aureli F. Aversion to violation of expectations of food distribution: the role of social tolerance and relative aversion in seven primate species. Behaviour. 2012;149(3–4):345–368. 10.1163/156853912X637833

[16] Schaub H. Testing kin altruism in long-tailed macaques *(Macaca fascicularis)* in a food-sharing experiment. Int J Primatol. 1996;17(3):445.

[17] Schwartz LP, Silberberg A, Casey AH, et al. Scaling outcome value with demand curves versus preference tests. Anim Cogn. 2016;19(3):631–641. 26908005 10.1007/s10071-016-0967-4 PMC4826314

[18] HREC (Health Research Ethics Committee). 2011a. National guidelines on health research ethics. Ministry of Health, Republic of Indonesia.

[19] HREC (Health Research Ethics Committee). 2011b. Teaching Guide Book for Ethics on Health Research. Ministry of Health, Republic of Indonesia.

[20] Brosnan SF. Nonhuman species’ reactions to inequity and their implications for fairness. Soc Justice Res. 2006;19(2):153–185.

[21] Brosnan SF, de Waal FBM. Evolution of responses to (un)fairness. Science. 2014;346(6207):1–7. 10.1126/science.1251776 PMC4451566 25324394

[22] Vinyard CJ, Cynthia CL, Doherty A, et al. Preference and consequences: a preliminary look at whether preference impacts oral processing in non-human primates. J Hum Evol. 2016;98:27–35. 27522640 10.1016/j.jhevol.2016.07.001

[23] Massen J, Luyten I, Spruijt B, et al. Benefiting friends or dominants: prosocial choices mainly depend on rank position in long-tailed macaques *(Macaca fascicularis)*. Primates. 2011;52(3):237–247. 21416218 10.1007/s10329-011-0244-8

[24] Brosnan SF, Talbot C, Ahlgren M, et al. Mechanisms underlying responses to inequitable outcomes in chimpanzees, *Pan troglodytes*. Anim Behav. 2010;79(6):1229–1237. 27011389 10.1016/j.anbehav.2010.02.019 PMC4801319

[25] Brosnan SF. A hypothesis of the co-evolution of cooperation and responses to inequity. Front Neurosci. 2011;5:43–55. 21519380 10.3389/fnins.2011.00043 PMC3077916

[26] Brosnan SF. Justice-and fairness-related behaviors in nonhuman primates. PNAS. 2013;110(2):10416–10423. 23754407 10.1073/pnas.1301194110 PMC3690609

[27] Brosnan SF, Hopper LM, Richey S, et al. Personality influences responses to inequity and contrast in chimpanzees. Anim Behav. 2015;101:75–87. 25722495 10.1016/j.anbehav.2014.12.019 PMC4337034

[28] Cronin KA, Snowdon CT. The effects of unequal outcome distributions on cooperative problem solving by cotton-top tamarins *(Saguinus oedipus)*. Anim Behav. 2008;75(1):245–257. 19122748 10.1016/j.anbehav.2007.04.032 PMC2390931

[29] Czoty PW, Gould RW, Nader MA. Relationship between social rank and cortisol and testosterone concentrations in male cynomolgus monkeys *(Macaca fascicularis)*. J Neuroendocrinol. 2009;21(1):68–76. 19094095 10.1111/j.1365-2826.2008.01800.x PMC2709846

[30] Sussman AF, Ha JC, Bentson KL, et al. Temperament in rhesus, longtailed, and pigtailed macaques varies by species and sex. Am J Primatol. 2013;75(4):303–313. 23225368 10.1002/ajp.22104 PMC3581757

[31] De Waal FBM. Food transfers through mesh in brown capuchins. J Comp Psychol. 1997;111(4):370–378. 9419882 10.1037/0735-7036.111.4.370

[32] Jaeggi AV, Stevens JM, Van Schaik CP. Tolerant food-sharing and reciprocity is precluded by despotism among bonobos but not chimpanzees. Am J Phys Anthropol. 2010;143(1):41–51. 20310060 10.1002/ajpa.21288

[33] Wittig RM, Crockford C, Deschner T, et al. Food-sharing is linked to urinary oxytocin levels and bonding in related and unrelated wild chimpanzees. Proc Royal Soc Biol Sci. 2014;281(1778):20133096. 10.1098/rspb.2013.3096 PMC3906952 24430853

[34] Mustoe AC, Harnisch AM, Hochfelder B, et al. Inequity aversion strategies between marmosets are influenced by partner familiarity and sex but not by oxytocin. Anim Behav. 2016;114:69–79. 27019514 10.1016/j.anbehav.2016.01.025 PMC4802974

[35] Bräuer J, Call J, Tomasello M. Are apes inequity averse? New data on the token-exchange paradigm. Am J Primatol. 2009;71(2):175–181. 19021260 10.1002/ajp.20639

[36] Parsons LH, Hurd YL. Endocannabinoid signalling in reward and addiction. Nat Rev Neurosci. 2015;16(10):579–594. 26373473 10.1038/nrn4004 PMC4652927

[37] Mendoza RB, van Coeverden CR, Schultz W. A neuronal reward inequity signal in primate striatum. J Neurophysiol. 2016;115(1):68–79. 26378202 10.1152/jn.00321.2015 PMC4760476

[38] Roll ET. Reward systems in the brain and nutrition. Annu Rev Nutr. 2016;36(14):1–14. 27146018 10.1146/annurev-nutr-071715-050725

[39] Paulus M. Children’s inequity aversion depends on culture: a cross-cultural comparison. J Exp Child Psychol. 2015;132:240–246. 25626404 10.1016/j.jecp.2014.12.007

[40] Raihani NJ, McAuliffe K. Human punishment is motivated by inequity aversion, not a desire for reciprocity. Biol Lett. 2012;8(5):802–804. 22809719 10.1098/rsbl.2012.0470 PMC3441003

